# Lipreading in speech-language-hearing forensic analysis: perspectives and challenges

**DOI:** 10.1590/2317-1782/e20250046en

**Published:** 2025-12-01

**Authors:** Renata Christina Vieira, Letícia Troian de Souza

**Affiliations:** 1 Universidade do Porto - Porto, Portugal.; 2 Fundação de Saúde Cristo Rei - Matipó (MG), Brasil.

Dear Editors-in-Chief,

The speech-language-hearing (SLH) sciences have been expanding their reach in the legal field through SLH forensic analysis. Lipreading is a promising technique whose applications, potential, and limitations merit discussion. It is used to identify words uttered by a speaker based on the movements of the articulatory organs, consistent with the context of analysis^(1,2)^. Although lipreading identifies only 50% of speech, it may be the only viable source of evidence in certain cases, which justifies its use. This technique seeks to understand speech through the visual analysis of lip movements and facial expressions, an area of ​SLH ​expertise that integrates knowledge of articulation, phonetics, language, and orofacial dynamics^(3,4)^. In SLH, this skill helps to validate testimonies and clarify situations that require investigation and depend on understanding the speech of someone who has been filmed and whose audio cannot be verified, only the image. The reliability of the analysis depends on several factors, such as the quality of the images and the expert's mastery of phonetic and linguistic knowledge. Lipreading expertise lies at the crossroads between forensic phonetics and forensic linguistics, integrating concepts from both fields to provide a more accurate interpretation of speech based on visual-perceptual evaluation. There are several studies on lipreading, but they typically focus on assisting deaf individuals, focusing on improving speech comprehension through visual training^(5-8)^, and on the possibility of automatic lipreading. The literature on lipreading highlights the importance of its use in investigations and in supporting law enforcement and argues for its relevance to the legal field^(9)^.

Although lipreading has been studied to assist deaf people, its forensic use has not been adequately addressed. Lipreading expertise can play an important role in investigations and legal support, but it is also important to analyze its limitations and potential.

## LIMITATIONS

The following limitations stand out in forensic lipreading: admissibility, it lacks robust methodology, leading to potential uncertainty in the analysis results. SLH sciences contribute to the initial stage of the forensic analysis, verifying whether the samples meet the minimum criteria for producing evidence^(10)^. Integrity, quantity, and quality are the elements of this stage that most effectively affect the use of lipreading. Integrity refers to the analysis of the material to determine whether there has been any alteration, damage, or modification to the video. The quantity aspect of lipreading relates to the number of frames in the image, as poor image quality affects the visualization of articulatory movements. Quality in the context of lipreading is related to resolution, brightness, focus, camera positioning, and the quality of the equipment. Inadequate quality affects image clarity, making the task impossible. Furthermore, it's important to ensure the face is visible; wearing hats and masks can impede visualization of articulatory movements. Another aspect that limits the technique's use is the lack of a robust methodology, established protocols, and studies in the field, reducing the reliability of expert analyses. The absence of a method can lead the expert to biased interpretations, subjecting them to error. This brings us to the third limiting factor in the technique's use: the potential uncertainty in the analysis results. As mentioned previously, lipreading allows us to visualize 50% of speech, as it can only capture phonemes produced in the anterior part of the vocal tract. Furthermore, some phonemes are homorganic (i.e., produced similarly), which prevents visual distinction between them. It's also important to consider that lipreading doesn't capture important information for speech comprehension, such as prosody and situational context, which are crucial for fully understanding what is being said.

## POTENTIALS

The strengths of lipreading include the knowledge of hypotheses and context, the expert’s comprehensive knowledge of phonetics and linguistics, the use of videos of the suspect as standard material, and partnership with lipreaders. Understanding hypotheses allows one to validate what witnesses claim to have heard and verify whether this is consistent with the observed lip movements^(11)^. This knowledge helps reduce ambiguities by comparing what was said with the allegations, which makes the analysis more accurate. Knowledge of context facilitates speech interpretation and allows one to predict more expected words or phrases, reducing the margin of error and increasing the reliability of lipreading. Phonetic knowledge is important for identifying visible and invisible sounds, facilitating the prediction of what was said by observing lip movements^(12-15)^. Linguistic knowledge involves understanding grammatical structure and vocabulary, helping to correctly associate lip movements with words^(12,16,17)^. Proficiency in auditory-perceptual evaluation contributes to the identification of a speaker's vocal characteristics, especially vocal tract adjustments^(18)^, improving lipreading accuracy. Furthermore, geolinguistic knowledge facilitates the identification of regional variations, considering differences in pronunciation and local expressions, which enhances the analysis. It is important to investigate the individual's background, including their birthplace and place of residence, as these factors can influence how speech is articulated and perceived. Studying videos of the suspect speaking allows us to understand their vocal and linguistic profile and identify individual articulation patterns^(19)^. If access to videos of the suspect is not possible, the court may request the collection of standard material. Regarding partnerships with lipreaders, qualitative studies largely depend on the investigator's analytical (subjective) ability. Reliability checks can be performed^(20)^, meaning another researcher can analyze the data and verify whether the results agree or disagree. Partnering with individuals skilled in lipreading can validate interpretations and strengthen the analysis, as they can make pertinent observations about lip movements and help confirm hypotheses.

## FINAL CONSIDERATIONS

Lipreading is an important SLH analytical tool, essential for validating testimonies and analyzing situations where audio from video recordings is inaudible or nonexistent. As a technique, it requires the SLH pathologist to have in-depth knowledge of speech articulation, orofacial dynamics, and contextual language analysis, integrating phonetic and linguistic aspects to ensure greater accuracy.
