# Supplementary material for: Lipreading in speech-language-hearing forensic analysis: perspectives and challenges
Source: Codas. 2025 Dec 1;37(6):e20250046. doi: 10.1590/2317-1782/e20250046en (PMC12704605; doi:10.1590/2317-1782/e20250046en)
Supplement: Translation

# Translation: A técnica de leitura labial na perícia fonoaudiológica: perspectivas e desafios

**DOI:** 10.1590/2317-1782/e20250046pt

**Authors:** Renata Christina Vieira, Letícia Troian de Souza

**Affiliations:** 1 Universidade do Porto - Porto, Portugal.; 2 Fundação de Saúde Cristo Rei - Matipó (MG), Brasil.

Prezadas editoras-chefes,

A Fonoaudiologia vem expandindo sua atuação no contexto jurídico por meio da Perícia Fonoaudiológica, destacando a leitura labial como uma técnica promissora, cujas aplicações, potencialidades e limitações merecem discussão. A leitura labial é uma técnica aplicada para a identificação de palavras emitidas pelo falante por intermédio da compreensão dos movimentos dos órgãos articulatórios, condizente com o contexto de análise^(1,2)^. Embora a leitura labial permita identificar apenas 50% da fala, em certos casos, pode ser a única fonte viável de prova. Por tal fato justifica-se o uso da técnica. A leitura labial é uma técnica que busca o entendimento da fala por meio da análise visual dos movimentos labiais e expressões faciais, área de expertise da Fonoaudiologia, que integra conhecimentos sobre articulação, fonética, linguagem e dinâmica orofacial^(3,4)^. No campo pericial fonoaudiológico, essa habilidade auxilia na validação de depoimentos e no esclarecimento de situações que precisam ser investigadas e dependem do entendimento da fala de alguém que foi filmado e do qual não é possível verificar o áudio, apenas a imagem. A confiabilidade da análise depende de diversos fatores como a qualidade das imagens e o domínio dos conhecimentos fonéticos e linguísticos pelo especialista. A perícia de leitura labial está inserida em uma zona fronteiriça entre a Fonética Forense e a Linguística Forense, integrando conceitos de ambos os campos para fornecer uma interpretação mais precisa da fala sob o ponto de vista da análise perceptiva visual. Embora existam diversos estudos sobre leitura labial, estes costumam se ater ao auxílio a pessoas surdas, focando no aprimoramento da compreensão da fala por meio de treinamentos visuais^(5-8)^ e sobre a possibilidade de leitura labial automática. A literatura sobre leitura labial inclui a importância de seu uso em investigações e apoio a forças de segurança, além de defender a relevância deste uso para o campo jurídico^(9)^.

Embora a leitura labial tenha sido estudada no contexto de auxílio a pessoas surdas, seu uso forense não foi abordado da maneira necessária. A perícia de leitura labial pode ter um papel importante em investigações e no apoio ao campo jurídico, mas também faz-se importante analisar suas limitações e potencialidades.

## LIMITAÇÕES

No uso da técnica de leitura labial em contextos forenses, destacam-se como limitações: a admissibilidade, ausência de metodologia robusta e a possível incerteza nos resultados da análise. A admissibilidade no campo pericial fonoaudiológico corresponde a uma etapa inicial da perícia, na qual se verifica se as amostras atendem a critérios mínimos para a produção de prova^(10)^. Os elementos desta etapa que afetam de maneira mais efetiva o uso da leitura labial são: integridade, quantidade e qualidade. A integridade é o aspecto que se refere a análise do material para se averiguar se houve algum tipo de alteração, dano ou modificação do vídeo. O aspecto quantidade para a leitura labial se relaciona com a quantidade de frames da imagem, pois a baixa qualidade da imagem afeta a visualização dos movimentos articulatórios. A qualidade no contexto da leitura labial está relacionada a resolução, luminosidade, foco e posicionamento da câmera, além da qualidade do equipamento. Quando o elemento qualidade não está adequado, há influência na clareza da imagem, impossibilitando a tarefa. Além disso, é preciso observar se a face está visível, o uso de chapéus e máscaras podem impedir a visualização dos movimentos articulatórios. Outro aspecto que limita o uso da técnica é a ausência de metodologia robusta. A falta de protocolos e estudos consolidados na área diminui a confiabilidade das análises periciais. A ausência de um método pode levar o perito a interpretações com viés e, por isso, sujeita a erros. Este aspecto nos leva ao terceiro fator de limitação de uso da técnica, que é a possível incerteza nos resultados da análise. Como dito anteriormente, a leitura labial nos permite visualizar 50% da fala. Isso ocorre porque ela consegue captar apenas os fonemas produzidos na parte anterior do trato vocal. Além disso, existem fonemas que são produzidos de forma semelhante, ou seja, são homorgânicos, o que impede a distinção visual entre eles. Também é preciso levar em consideração que a leitura labial não captura informações importantes para a compreensão da fala, como a prosódia e o contexto situacional, que são fundamentais para a compreensão total do que é dito.

## POTENCIALIDADES

No uso da leitura labial, destacam-se como potencialidades o conhecimento de hipóteses e do contexto, que o perito entenda profundamente sobre Fonética e Linguística, o uso de vídeos do suspeito como material padrão e parcerias com leitores labiais. Conhecer hipóteses permite validar o que as testemunhas afirmam ter ouvido e verificar se isso é compatível com os movimentos labiais observados^(11)^. Esse conhecimento ajuda a reduzir ambiguidades, permitindo uma comparação entre o que foi dito e as alegações feitas, o que torna a análise mais precisa. Já o conhecimento do contexto facilita a interpretação da fala e permite prever palavras ou frases mais esperadas, o que reduz a margem de erro e aumenta a confiabilidade da leitura labial. O conhecimento fonético é importante para identificar sons visíveis e invisíveis, facilitando a previsão do que foi dito com a observação dos movimentos labiais^(12-15)^. O conhecimento linguístico envolve entender a estrutura gramatical e o vocabulário, ajudando a associar corretamente os movimentos labiais às palavras^(12,16,17)^. Ter proficiência em análise perceptivo-auditiva contribui para a identificação de características vocais do falante, principalmente ajustes de trato vocal^(18)^, melhorando a precisão da leitura labial. Além disso, os conhecimentos geolinguísticos facilitam a identificação de variações regionais, considerando diferenças de pronúncia e expressões locais, o que aprimora a análise. Cabe investigar o histórico do indivíduo, onde nasceu e viveu, já que esses fatores podem influenciar a forma como a fala é articulada e percebida. O estudo de vídeos do suspeito falando permite entender seu perfil vocal e linguístico, e identifica padrões individuais de articulação^(19)^. Caso não seja possível o acesso a vídeos do suspeito, pode ser solicitada pela Justiça a coleta de material padrão. Sobre parcerias com leitores labiais, os estudos qualitativos dependem, em grande parte, da capacidade analítica do investigador (subjetiva). É possível realizar verificações de fiabilidade^(20)^, isso significa que um outro investigador pode analisar os dados e, em seguida, verificar se os resultados estão de acordo ou desacordo. As parcerias com indivíduos que têm habilidade em leitura labial podem validar interpretações e fortalecer a análise, pois esses leitores labiais podem oferecer observações pertinentes sobre os movimentos labiais e ajudar a confirmar hipóteses.

## CONSIDERAÇÕES FINAIS

A leitura labial representa uma importante ferramenta de análise na Perícia Fonoaudiológica, sendo essencial para a validação de depoimentos e a análise de situações em que o áudio de gravações em vídeo é inaudível ou inexistente. Como técnica, exige do perito fonoaudiólogo um profundo conhecimento sobre articulação da fala, dinâmica orofacial e análise contextual da linguagem, integrando aspectos fonéticos e linguísticos para garantir maior precisão nos resultados.
